# Phytochemical Analysis, Antimalarial Properties, and Acute Toxicity of Aqueous Extracts of Trisamo and Jatu-Phala-Tiga Recipes

**DOI:** 10.1155/2023/6624040

**Published:** 2023-09-15

**Authors:** Arisara Phuwajaroanpong, Prapaporn Chaniad, Walaiporn Plirat, Atthaphon Konyanee, Abdi Wira Septama, Chuchard Punsawad

**Affiliations:** ^1^Department of Medical Sciences, School of Medicine, Walailak University, Nakhon Si Thammarat 80160, Thailand; ^2^Research Center in Tropical Pathobiology, Walailak University, Nakhon Si Thammarat 80160, Thailand; ^3^Research Center for Pharmaceutical Ingredient and Traditional Medicine, National Research and Innovation Agency (BRIN), Cibinong Science Center, Bogor 16915, Indonesia

## Abstract

Drug resistance remains a significant problem that threatens antimalarial drug treatment. Hence, the challenge is to find new effective antimalarial drugs. Based on our previous study, aqueous extracts of trisamo (TSM) and jatu-phala-tiga (JPT) had good *in vitro* antimalarial activities, and these recipes contain multiple beneficial pharmacological effects that could be useful for malaria therapy. Therefore, this study aimed to investigate the antimalarial activity and toxicity of the aqueous extracts of TSM and JPT in mouse models. The aqueous extractions were carried out using the decoction method. Compound identification was conducted using LC-QTOF-MS analysis. The antimalarial activities of TSM and JPT at doses 200, 400, and 600 mg/kg were evaluated against *Plasmodium berghei* ANKA infection using a four-day suppressive test. The toxic effects of oral administration of the extracts at 2 g/kg dose were determined using an acute toxicity test. The chemical constituents of TSM contained 83 compounds, whereas JPT contained 84 compounds. All doses of the extracts exhibited a significant suppression (*p* < 0.05) of the parasite compared to the negative control in a four-day test. The maximum activities were observed at 600 mg/kg dose with 67.02% suppression for TSM and 79.34% for JPT, followed by 400 mg/kg dose (57.63% for TSM and 64.79% for JPT) and then 200 mg/kg dose (52.35% for TSM and 54.46% for JPT). In addition, there were no significant differences (*p* < 0.05) in the RBC, MCV, and MCH levels of mice receiving JPT extract compared to the uninfected control. The WBC level of mice receiving 400 and 600 mg/kg of TSM, and 200 and 400 mg/kg of JPT, was significantly (*p* < 0.05) lower than the infected control, and the extracts did not significantly prevent the loss of platelets. For the acute toxicity test, there were no signs of toxicity or deaths in mice, and there were no differences in the histology, weight, or enzyme biochemistry of the liver and kidney between the extract and vehicle groups. However, the platelet count in the extract-treated mice was significantly higher than that in the control group. In conclusion, this study suggests that aqueous extracts of TSM and JPT have potent antimalarial activities and could be promising as new candidates for antimalarial drug development.

## 1. Introduction

Malaria remains one of the most serious illnesses in tropical and subtropical regions. It is a vector-borne disease caused by obligate intracellular *Plasmodium* parasites and is transmitted through the bite of infected female Anopheles mosquitoes [1]. Five species of Plasmodium, *P. falciparum*, *P. vivax*, *P. malariae*, *P. ovale*, and *P. knowlesi*, can cause diseases in humans [2]. Malarial paroxysm is a distinct clinical feature of the disease caused by the rupture of infected red blood cells (RBCs) [2]. Common symptoms include fever, chills, and headaches, whereas the major complications of severe malaria include cerebral malaria, pulmonary edema, acute renal failure, severe anemia, bleeding, liver injury, and death [2]. According to the World Malaria Report of 2022, the estimated number of deaths globally was 619,000, and 247 million cases were reported [1]. The African region of the World Health Organization (WHO) has the highest malaria burden, accounting for approximately 95% of the global cases [1]. However, the case incidence has dropped since 2000 before increasing in 2020 due to a service interruption during the COVID-19 pandemic. Although effective treatment is a key factor in combating this disease, drug resistance poses a significant risk to the control and elimination of malaria [3]. After the emergence of chloroquine resistance in the 1960s, the WHO recommended artemisinin-based combination therapy (ACT) as the first-line treatment for uncomplicated malaria [4], which included artemether-lumefantrine (AL), artesunate-amodiaquine (AS-AQ), artesunate-mefloquine (AS-MQ), artesunate-sulfadoxine-pyrimethamine (AS-SP), dihydroartemisinin-piperaquine (DHA-PPQ), and artesunate-pyronaridine (AS-PY) combinations [3, 4]. The various treatments were determined based on the areas of resistance. Currently, the emergence of partial artemisinin resistance is of great concern and has been observed in countries in the WHO African region and the Greater Mekong Subregion (GMS) [1]. A treatment failure rate of >10% for AL in Burkina Faso and Uganda and for DHA-PPQ in Burkina Faso has been observed [1]. In GMS, mutations associated with SP resistance have been observed; hence, the failure of AS-SP could be of concern [1]. In addition, a high prevalence of mutations associated with partial artemisinin resistance was found in Myanmar and Thailand, and high rates of DHA-PPQ plus primaquine treatment failure were found in Sisaket Province, Thailand, which led the province to change its first-line drug to AS-PY in 2020 [1]. Novel strategies are needed to eradicate malarial parasites to overcome the emergence of drug-resistant parasites. Utilizing traditional medicine is one of several interesting ideas, especially polyherbal or herbal recipes, owing to their positive effects as a result of synergistic interactions [5]. Our previous study on the *in vitro* antiplasmodial properties of aqueous and ethanolic extracts of ten herbal traditional recipes reported that aqueous extracts of trisamo (TSM) and jatu-phala-tiga (JPT) exhibited good antimalarial activities [6]. Both are traditional herbal recipes that have been used for several centuries in Thailand [7]. The TSM is composed of three *Terminalia* species: *Terminalia bellirica* (Gaertn.) Roxb., *Terminalia chebula* (Roxb. ex DC.), and *Terminalia arjuna* (Roxb. ex DC.). Trisamo means “three” (tri-) fruits of *Terminalia* species (-samo), and these plants belong to the family *Combretaceae* [7]. The common names of *T. bellirica*, *T. chebula*, and *T. arjuna* are beleric myrobalans, chebulic myrobalans, and arjuns, respectively [7]. TSM is indicated for promoting good general health and relieving abdominal bloating and is also used as an antipyretic, expectorant, and rejuvenator [8]. Several biological benefits of the ingredients in the TSM recipe have been reported which include antipyretic, antibacterial, antioxidant, anti-inflammatory, antihyperglycemic, anticlastogenic, immunomodulatory, analgesic, radioprotective, gastrointestinal motility-promotion, cardioprotective, antiaging cytoprotective, anticancer, antidiabetic, wound-healing, and antinociceptive properties [8–11]. The meaning of JPT corresponds to the benefits of four fruits: Jatu means “four,” phala means “fruits,” and tiga means “benefits” or “usefulness.” The four fruit ingredients include *Phyllanthus emblica* Linn. (*P. emblica*), *T. bellirica*, *T. chebula*, and *T. arjuna*. Indian gooseberry or *P. emblica* belongs to the family *Euphorbiaceae* and is commonly used in Ayurvedic systems for its many beneficial characteristics such as antidiabetic, antimicrobial, anti-inflammatory, and antiaging properties [7, 12]. JPT is well known for its antioxidant activity, and it is used as an antipyretic, laxative, stomachic, colon cleanser, detoxifying agent, health promotion agent, and rejuvenator in Thai traditional medicine [13, 14]. Scientific evidence has revealed that JPT has antimutagenic, cardioprotective, radioprotective, hepatoprotective, anti-inflammatory, and antiobesity properties [15, 16]. In addition, the TSM and JPT recipes consist of numerous secondary metabolites such as flavones, alkaloids, phenols, tannins, coumarin, terpenoids, glycosides, and saponins [17–19]. Several classes of phytoconstituents from natural products are responsible for their antimalarial activity [20]. Alkaloids, including terpenoidal, quinolone, and isoquinoline alkaloids, were identified with promising antimalarial activity [21]. The antimalarial action of plant flavonoids is believed to act by inhibiting fatty acid biosynthesis and the influx of L-glutamine-myoinositol in the infected red blood cells [22]. Terpene and coumarin derivatives have been reported to have potent antimalarial activities [23, 24]. Regarding the abovementioned, the phytoconstituents deposited in the TSM and JPT recipes may provide great potential for antimalarial activities. Thus, this study aimed to investigate the antimalarial activity and toxicity of TSM and JPT in a mouse model.

## 2. Materials and Methods

### 2.1. Management and Preparation of TSM and JPT Recipes

*T. bellirica*, *T. chebula*, *T. arjuna*, and *P. emblica* were bought at a Thai pharmacy store in the southern Thai province of Nakhon Si Thammarat's Muang District. The morphological identification of plants was confirmed by a botanist, and the deposited specimens SMD074002003 (*T. bellirica*), SMD070006007 (*T. chebula*), SMD070006002 (*T. arjuna*), and SMD209003007 (*P. emblica*) were at Walailak University in Thailand's School of Medicine's Department of Medical Sciences. The fruits were dried for 3 days in an oven (Memmert Model SFE 600, Schwabach, Germany) after being washed with tap water. Each fruit was ground using a herb grinder (Taizhou Jincheng Pharmaceutical Machinery Co., Ltd., Model: SF, Jiangsu, China). The TSM and JPT recipes were prepared according to Thai herbal pharmacopeia [7, 25]. The TSM recipe was prepared by mixing *T. bellirica*, *T. chebula*, and *T. arjuna*, in a 1 : 1 : 1 ratio, and the JPT recipe was prepared by mixing *T. bellirica*, *T. chebula*, *T. arjuna*, and *P. emblica* in a 1 : 1 : 1 : 1 ratio.

### 2.2. Aqueous Extraction Method

Aqueous extractions of TSM and JPT were performed using the decoction method [26, 27]. For each recipe, 60 g of plant material suspended in 600 mL of water was extracted by boiling for 30 min. Then, filtration through filter paper (Whatman, Buckinghamshire, England) was used to separate the liquid from the marc. Subsequently, the marc was re-extracted twice by boiling in 600 mL of water for 30 min. The rotary evaporator (Rotavapor, Buchi, China) was used to concentrate the combined filtrate at 45 rpm and 45°C. Then, the extract was dried at −89°C in a freeze-drying apparatus (Martin Christ, Germany). The crude extract was weighed, and the yield was determined as follows:

(1) $$\begin{array}{c} \text{Percentage yield}=\frac{\text{weight of crude extract}}{\text{initial weight of herbal recipe}}\times 100\text{.} \end{array}$$

### 2.3. Compound Identification Using Liquid Chromatography-Quadrupole Time-of-Flight Mass Spectrometry (LC-QTOF-MS) Analysis

Chromatographic separations were accomplished according to our previous study [6]. The temperature in the column was fixed at 25°C. The mobile phase A was made up of 0.1% formic acid in water, and mobile phase B was made up of acetonitrile. A flow rate of the mobile phase was 0.20 mL/min, and the injection volume was 2 *μ*L. The MS conditions involved an electrospray ionization (ESI) probe in negative mode with a scanning range of 100−1,200 m/z. Agilent MassHunter Workstation Software V8 was used to process the data. Compound identification was based on the similarity score, which was achieved by matching the retention times and mass data of an unknown compound to the reference spectra in a METLIN mass spectra library (Agilent Technologies). A similarity score of >90% was employed to identify the compounds deposited in the extracts.

### 2.4. Animals and Management

Male ICR mice that were 6–8 weeks old were obtained from Nomura Siam International Co., Ltd., in Bangkok, Thailand. All mice were acclimatized for 1 week under strictly hygienic laboratory conditions. Housing settings included cycling light and dark for 12 hours each cycle, with a regulated room temperature of 23 ± 2°C and humidity levels of 50−70%. Mice were given unlimited access to food pellets and clean drinking water.

### 2.5. Testing for Antimalarial Activity

The antimalarial activity was evaluated using Peters' 4-day suppressive test [28]. The rodent malaria parasite, *P. berghei* ANKA strain, was provided by Thomas F. McCutchan and obtained from BEI Resources, NIAID, NIH. Inoculation was initiated by intraperitoneal (IP) injection of 0.2 mL of infected blood into the donor mice. Once the parasitemia level reached 20–30%, blood was collected to infect recipient mice. To evaluate the *in vivo* antimalarial activity against early infection, 40 mice received IP injection of 0.2 mL of 1 × 10^7^*P. berghei* infected cells, whereas five mice were injected with 0.2 mL of normal saline solution as uninfected controls. Forty infected mice were randomly divided into eight groups (five mice per group), which included negative (received phosphate-buffered saline (PBS)) and positive (received 25 mg/kg chloroquine) control groups, and six experimental groups (received TSM or JPT). Aqueous extracts of TSM and JPT were administered at doses 200, 400, and 600 mg/kg. Oral administration was started at 3 h and then at 24, 48, and 72 h post-infection. On day 4, all mice were anesthetized by inhalation of 2% isoflurane in oxygen and euthanized immediately after blood collection by cardiac puncture. Thin blood smears were made from blood samples to determine the percentage of parasitemia. In addition, blood samples were used to determine changes in hematological parameters, including red blood cell (RBC) count, hemoglobin (HGB), hematocrit (HCT), mean cell volume (MCV), mean corpuscular hemoglobin concentration (MCHC), mean corpuscular hemoglobin (MCH), platelet (PLT) count, and white blood cell (WBC) count. Hematological analysis was carried out with an automatic AU480 chemistry analyzer (Beckman Coulter, USA). Parasitemia was monitored by Giemsa-stained thin blood smears, which were then viewed under a light microscope (Olympus CX31, Model CX31RBSFA, Tokyo, Japan) with oil immersion (100× magnification). The following formula was used to obtain the parasitemia and suppression percentages:

(2) $$\begin{array}{c} \%\text{parasitemia}=\frac{\text{number of parasitised red cells}}{\text{number of total red blood cells}}\times 100\text{,}\\ \%\text{suppression}=\frac{\left(\text{mean parasitemia in negative group}-\text{mean parasitemia in experimental group}\right)}{\text{mean parasitemia in negative group}}\times 100\text{.} \end{array}$$

### 2.6. Acute Toxicity Test

This test was performed in accordance with the Organization for Economic Cooperation and Development (OECD) guideline No. 425 [29] according to a previously described method [6, 26, 30]. Three groups of five mice each were formed from a total of fifteen mice. PBS was used as the vehicle control for Group I. TSM and JPT were administered at a dose of 2 g/kg to Groups II and III, respectively. All mice were weighed before receiving the extract or PBS. To assess the toxicity of the extracts, 2 g/kg of the extract was administered after the mice had fasted for 3 h. The mice were observed immediately after feeding and then carefully observed for 30 min. Behavioral changes, signs of toxicity, and mortality were observed twice daily for 14 days. On day 14, all mice were weighed, anesthetized with 2% isoflurane, and euthanized through cardiac puncture. Blood samples were collected from the heart for hematological and biochemical analyses. The liver and kidneys were removed and weighed to determine the relative organ weights. Relative organ weight was calculated using the following formula; thereafter, the organs were used for histopathological analysis.

(3) $$\begin{array}{c} \text{Relative organ weight}=\frac{\text{organ weight}}{\text{body weight}}\times 100\text{.} \end{array}$$

### 2.7. Hematological, Biochemical, and Histopathological Assessment in Acute Toxicity Test

Blood was collected into two types of tubes including EDTA and serum clot activator tubes. Blood in EDTA tubes was used for the hematological analysis (RBC, HGB, HCT, MCV, MCHC, MCH, PLT, and WBC), whereas serum were used for evaluation of renal and hepatic functions, including aspartate aminotransferase (AST), alanine aminotransferase (ALT), alkaline phosphatase (ALP), blood urea nitrogen (BUN), and creatinine. Blood samples were analyzed using an AU480 chemistry analyzer (Beckman Coulter, USA). For histopathological assessment, the liver and kidneys were fixed in 10% formalin, and hematoxylin and eosin staining was performed as previously described [31–33].

### 2.8. Statistical Analysis

SPSS for Microsoft Windows (version 17.0; IBM, Armonk, NY, USA) was used to conduct the statistical analyses. The mean ± standard error of the mean (SEM) is used to express all data. The data were examined for normality of distribution before being subjected to a one-way analysis of variance (ANOVA), with a significance level of *p* < 0.05.

## 3. Results

### 3.1. Percentage Yield of TSM and JPT Recipes

The percentage yield of the aqueous extracts of JPT (40.12) was slightly higher than that of TSM (39.62). The TSM appeared as a dark brown solid, and JPT was a light brown crumbly solid.

### 3.2. Compound Composition of TSM and JPT Detected by LC-QTOF-MS Analysis

**Figure 1:**
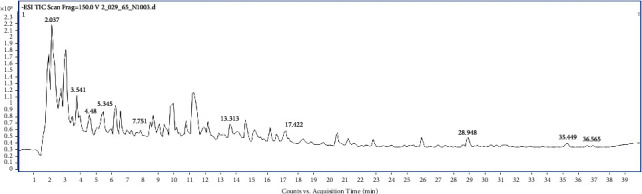
Full-scan chromatogram of TSM recipe.

**Figure 2:**
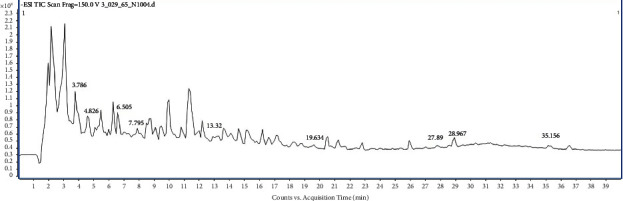
Full-scan chromatogram of JPT recipe.

The compounds in the TSM and JPT extracts were tentatively identified using LC-QTOF-MS analysis. Tables 1 and 2 show the compounds found in the aqueous extracts of TSM and JPT, respectively. The chemical constituents of TSM contained 83 compounds, whereas JPT contained 84 compounds. Figures 1 and 2 show the peak chromatograms of TSM and JPT, respectively.

### 3.3. Effects of the Extracts on Percentage Parasite Suppression

Percentage parasitemia and effects of crude extracts on the percentage suppression of *P. berghei* infection are shown in Table 3. The standard drug administered at a concentration of 25 mg/kg eliminated 100% of the blood-stage parasites. Administration of TSM and JPT extracts exhibited significant (*p* < 0.05) dose-dependent percentage parasite suppression compared to the negative control, with mean suppression percentage ranges 52.35–67.02% for TSM and 54.46–79.34% for JPT. In addition, percentage suppression at all doses of TSM was significantly lower than that in the chloroquine group (*p* < 0.05), whereas the percentage suppression at 600 mg/kg JPT showed no significant difference (*p* < 0.05) compared to chloroquine administration.

### 3.4. Effects of the Extracts on Hematological Changes in 4-Day Suppressive Test

**Figure 3:**
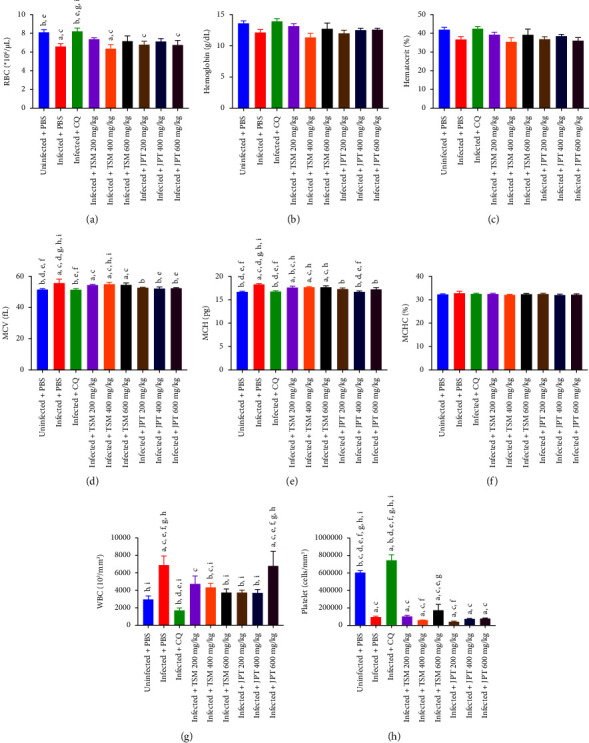
Effects of TSM and JPT on hematological parameters in the 4-day suppressive test: (a) RBC count, (b) hemoglobin levels, (c) hematocrit levels, (d) MCV levels, (e) MCH levels, (f) MCHC levels, (g) WBC count, and (h) platelet count. Data are presented as the mean ± SEM (*n* = 5 per group). There were statistically significant differences at *p* < 0.05, ^a^compared with uninfected mice, ^b^compared with negative control group receiving PBS, ^c^compared with the positive control group receiving CQ, ^d^compared with TSM 200 mg/kg, ^e^compared with TSM 400 mg/kg, ^f^compared with TSM 600 mg/kg, ^g^compared with JPT 200 mg/kg, ^h^compared with JPT 400 mg/kg, and ^i^compared with JPT 600 mg/kg.

The results of the hematological changes are shown in Figure 3. The indices of uninfected mice were used to represent hematologic reference values at the normal levels. Hematological alterations between normal and infected controls showed significant differences in RBC, MCV, and MCH levels (*p* < 0.05), while hemoglobin, hematocrit, and MCHC levels were not significantly different among all groups. Mice administered a standard drug demonstrated significantly higher RBC levels (*p* < 0.05), but MCV and MCH levels were significantly lower (*p* < 0.05) than those in the infected controls. TSM administered at a dose of 400 mg/kg revealed a significant decrease in RBC (*p* < 0.05), but JPT administration did not show a significant decrease in RBC compared with uninfected mice and those administered chloroquine. The differences between MCV and MCH levels showed that mice receiving JPT at all doses exhibited significantly (*p* < 0.05) lower levels than the infected control, and no difference was observed when compared to the chloroquine group. The MCV and MCH levels in the TSM group were significantly (*p* < 0.05) higher than those in the uninfected and chloroquine groups. Administration of 400 mg/kg TSM resulted in a significant increase in MCV, compared to 400 and 600 mg/kg of JPT, and all doses of TSM resulted in a significant difference in the MCH level compared to 400 mg/kg of JPT. The platelet counts of infected mice and mice that received the extracts were significantly (*p* < 0.05) lower than those of the normal control. Compared to the infected control, only the positive control group showed a significant increase in platelet counts (*p* < 0.05). In addition, chloroquine also produced a significant (*p* < 0.05) decrease in the WBC count compared to that in infected mice. The extracts did not prevent a significant (*p* < 0.05) loss of WBC compared with chloroquine. TSM (400 and 600 mg/kg) and JPT (200 and 400 mg/kg) were significantly decreased when compared to the infected control.

### 3.5. Clinical Observations, Analysis of Bodyweight, and Organs' Weights in Acute Toxicity Test

Oral administration of TSM or JPT at a dose of 2 g/kg in mice did not produce any significant changes in clinical signs compared to the vehicle control. There were general physical and behavioral appearances such as bright eyes, erect ears, normal body posture, and grooming. Signs of toxicity, such as diarrhea, tremors, convulsions, ataxia, or unusual behaviors, were not observed throughout 14 days. No mortality occurred during the experiment; therefore, the mean lethal dose (LD_50_) of the aqueous extract of TSM and JPT administered via the oral route was higher than 2 g/kg. When compared to the vehicle control, the actual body weight, percentage of body weight change, and relative organ weights of the liver and kidney of mice administered that the extracts showed no significant difference (*p* > 0.05) (see in Table 4).

### 3.6. Effects of the Extracts on Hematological and Biochemical Changes in Acute Toxicity Test

**Figure 4:**
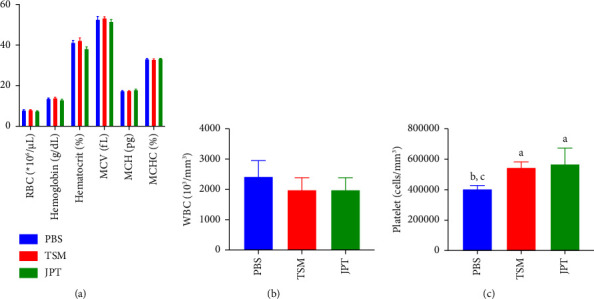
Effects of the extracts on hematological changes in acute toxicity test: (a) RBCs and related parameters, (b) WBC count, and (c) platelet count. All values are expressed as mean ± SEM (*n* = 5 per group). There were statistically significant differences at *p* < 0.05, ^a^compared with negative control, ^b^compared with 2 g/kg of TSM extract, and ^c^compared with 2 g/kg of JPT extract.

Hematological results revealed that the platelet counts of the TSM and JPT groups were significantly higher than that of the vehicle control group (see Figure 4). Biochemical parameters of the liver and kidney function tests are presented in Table 5. No significant differences in BUN, CREA, AST, ALT, or ALP levels were observed among the groups.

### 3.7. Effects of TSM and JPT on Histopathology in Acute Toxicity Test

**Figure 5:**
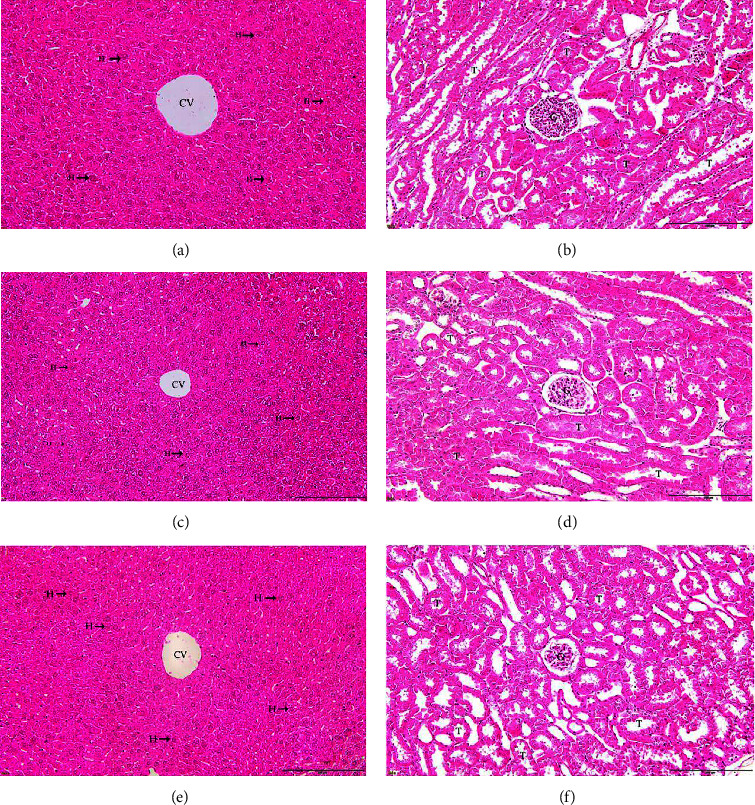
Histopathological micrograph of the liver and kidney of mice in the acute toxicity test. All images were acquired at 20× magnification. Bar = 20 *μ*m; CV: central vein; H hepatocyte; T renal tubule; G glomerulus. (a) Liver histology of control mice; (b) kidney histology of control mice; (c) liver histology of TSM-treated mice; (d) kidney histology of TSM-treated mice; (e) liver histology of JPT-treated mice; (f) kidney histology of JPT-treated mice.

Figure 5 shows the histopathological examination of the liver and kidneys in the acute toxicity test at a dose of 2 g/kg. Figures 5(a) and 5(b) show the normal structure of the liver and kidney histology, respectively, which were obtained from mice in the control group. In comparison, liver and kidney sections showed no differences between the control and mice treated with TSM and JPT. Liver sections (Figures 5(a), 5(c), and 5(e)) revealed normal hepatocytes without hepatic congestion, inflammatory cell infiltration, or sinusoidal dilatation. Kidney sections (Figures 5(b), 5(d), and 5(f)) showed unchanged glomeruli and renal tubules without vascular congestion.

## 4. Discussion

As antimalarial drug resistance has been a major problem in malaria control, effective vaccines are unavailable. Therefore, new treatments are urgently needed. Our previous report showed that aqueous extracts from TSM and JPT have potent antiplasmodial activity against *P. falciparum* [6]. As a result, the current study sought to assess the antimalarial properties and acute toxicity of aqueous extracts of TSM and JPT in mouse models.

For *in vivo* antimalarial testing, the antimalarial activities of TSM and JPT were investigated at 200, 400, and 600 mg/kg using a 4-day suppressive test. The highest average percentage parasite suppression of 600 mg/kg JPT was 79.34%, and the extracts at all doses significantly suppressed parasite growth compared with the infected control. However, only JPT at 600 mg/kg showed no significant difference (*p* < 0.05) compared to chloroquine, which may imply that the effect of JPT at 600 mg/kg is similar to that of chloroquine. The biological properties of plant extracts are known to be mediated by phytocomponents [34]. Based on the LC-MS analysis, our findings are consistent with those of previous studies. Chebulinic acid, gallic acid, ellagic acid, quinic acid, and luteolin were found in the fruits of *T. bellirica*, *T. chebula*, and *T. arjuna* extracts [8, 35, 36]. In addition, the phytocomponent of *P. emblica* was reported to have tannins such as chebulic acid, gallic acid, and punicalagin; flavonoids such as luteolin and quercetin derivatives; polyphenolics such as ellagic acid; and phenolics such as chebulinic acid [12, 37–39]. Ellagic acid has been reported to possess antioxidant, anti-inflammatory, antimutagenic, antiproliferative, and antimalarial properties [40, 41]. Chebulinic acid has been reported to have numerous biological activities, including antidiabetic, antifibrotic, anti-inflammatory, antitumor, antiatherogenic, antioxidant, antiulcer, hepatoprotective, and antiviral properties [42]. Quinic acid has an important antibacterial effect [43]. Accordingly, the compound described above, or the other compounds present in TSM and JPT might exert antimalarial activities through individual or synergistic effects. In addition, the antimalarial activity of the extracts in this study showed that the percentage parasite suppression of JPT was higher than that of TSM. The reason for this could be the extra ingredient from *P. emblica* in the JPT recipes. *P. emblica* is important in traditional medicinal systems, and its various pharmacological benefits have been reported, including antimicrobial, antioxidant, anti-inflammatory, antipyretic, antitusive, antiatherogenic, anticancer, antidiabetic, antiaging, cardioprotective, gastroprotective, nephroprotective, neuroprotective, chemopreventive, analgesic, and immunomodulatory properties [44]. Furthermore, JPT and its components have been reported to exhibit strong antioxidant activity [45]. Based on previous evidence, we suggest that *P. emblica* improves the antimalarial property of the extract.

Furthermore, this study investigated the effects of the extracts on hematological parameters during malaria infection because hematological abnormalities are considered a characteristic of malaria, especially RBCs [46]. The differences in RBC, MCV, and MCH between the uninfected and infected groups were significant, and chloroquine improved these blood parameters to normal ranges compared to the uninfected control. The reduction of RBCs in infected mice may be caused by the destruction or sequestration of RBCs, or reduction of RBC production in the bone marrow [9]. The significant increases in MCV and MCH were indicative of malaria-induced macrocytic anemia [47, 48]. The RBC count in the 400 mg/kg TSM group was significantly lower than that in the uninfected control. The MCV and MCH of all TSM extract doses differed significantly from those of the uninfected control. However, the RBC count, MCV, and MCH of mice that received JPT showed no significant differences compared to the uninfected control. This finding implies that only the JPT extract can prevent malaria-induced macrocytic anemia. The ability to improve RBC and related parameters may be due to the inhibitory effects of the drug and its extracts on parasite growth. WBC and related parameters play an important role in infectious diseases [46]. WBC responds to infectious agents, and thrombocytopenia is a common feature of Plasmodium infection, which is associated with several mechanisms, such as endothelial damage and isolated platelet consumption [46]. The platelet count of mice in the infected control group was significantly reduced, but the WBC count was increased, when compared with the uninfected control group. The extracts from TSM and JPT showed a significant and dramatic decrease in platelet count compared to the normal control and chloroquine. This could mean that extracts at doses 200, 400, and 600 mg/kg did not maintain platelet homeostasis during malaria infection. In terms of WBC parameters, TSM reduced the WBC count in a dose-dependent manner. This finding may indicate that a decrease in the WBC count is associated with a reduction in the percentage of parasites. Interestingly, 600 mg/kg JPT resulted in the highest WBC count and the highest percentage parasite suppression among the extracts. This result implies that the antimalarial activity of JPT at this dose may be attributed to the activation of immune cells, which is consistent with a previous study [12]. *P. emblica*, an ingredient in the JPT recipe, has been reported to exhibit the ability to enhance immunity [12]. Consequently, JPT extract not only exhibited stronger antimalarial activity but also exhibited greater maintenance of RBC parameters than the crude extract from TSM.

Although natural products have been used to treat several diseases since ancient times, the negative effects of plant products must be considered. According to the results of the toxicity study, there were no deaths or physical or behavioral changes for 14 days, thereby indicating that the LD_50_ of TSM and JPT was greater than 2 g/kg. TSM and JPT were classified as relatively low acute toxicity hazards in Category 5 according to the international system of chemical classification [49]. The effects of the extracts on changes in body weight and organ weight are sensitive indicators for general health status and organ damage in animals [50, 51]. We found no significant changes in body weight or organ weight when compared with the vehicle control at the endpoint. In addition, this study focused on the negative effects of the extracts on the hematological markers, biochemical enzymes, and pathology of the liver and kidney. For all blood parameters, only the platelet count was significantly increased in mice treated with TSM and JPT compared to the vehicle control. This result implies that the extract at a dose of 2 g/kg may have an effect on platelet enhancement activity, suggesting that this effect may be beneficial for improving thrombocythemia in blood diseases such as malaria. To ensure the safety of the extracts, the biochemical enzymes and pathology of the liver and kidney were assessed. The liver and kidneys play a dominant role in drug metabolism and elimination after ingestion. The liver is well known as the primary organ for parasite development in the pre-erythrocytic stages, resulting in stiffness of the infected liver cells [52]. Acute kidney injury is a well-known significant organ dysfunction caused by malaria infection, and hematological abnormalities are regarded as a key feature of malaria infection [46, 53]. There were no significant changes in biochemical enzymes and histology of the liver and kidneys. This finding implies that the extracts were not associated with nephrotoxicity or hepatotoxicity caused by herbal medicine. Regarding the results of the toxicity test, aqueous extracts of TSM and JPT showed clear evidence that the extracts were considered safe in mice when administered at 2 g/kg, and these extracts may provide great choices for antimalarial drug candidates because they are safe for major cells that can be damaged by the Plasmodium parasite, such as hepatocytes and RBCs.

## 5. Conclusions

This study demonstrates that aqueous extracts of TSM and JPT exert potent antimalarial activities against *P. berghei* and are considered safe for oral administration. Therefore, TSM and JPT should be considered as an alternative treatment for malaria. Further experiments should be conducted to test the antimalarial activity in nonhuman primates and in clinical trials.

## Figures and Tables

**Table 1:** Tentative identification of the chemical constituents from TSM by LC-QTOF-MS analysis.

No.	M/Z	RT (min)	Compounds	Formula	Molecular weight (g/mol)
1	283.2638	1.536	(+)-Isostearic acid	C_18_H_36_O_2_	284.2711
2	181.0718	1.812	D-Sorbitol	C_6_H_14_O_6_	182.0790
3	195.0507	1.837	D-Mannose	C_6_H_12_O_7_	196.0580
4	383.1194	1.912	Acetyl-maltose	C_14_H_24_O_12_	384.1266
5	173.0456	2.037	Shikimic acid	C_7_H_10_O_5_	174.0529
6	205.0355	2.037	(R)-2-Hydroxybutane-1,2,4-tricarboxylate	C_7_H_10_O_7_	206.0428
7	355.0312	2.789	(+)-Chebulic acid	C_14_H_12_O_11_	356.0384
8	169.0145	3.403	Gallic acid	C_7_H_6_O_5_	170.0217
9	243.0512	3.541	1-O-Galloylglycerol	C_10_H_12_O_7_	244.0584
10	651.1193	3.879	Chrysoeriol 4′,7-diglucuronide	C_28_H_28_O_18_	652.1266
11	325.0564	4.092	Fertaric acid	C_14_H_14_O_9_	326.0636
12	235.0245	4.242	2-Hydroxy-3-carboxybenzalpyruvate	C_11_H_8_O_6_	236.0319
13	191.0349	4.280	5,7-Dihydroxy-4-methylcoumarin	C_10_H_8_O_4_	192.0422
14	265.0352	4.480	2-O-p-Coumaroyltartronic acid	C_12_H_10_O_7_	266.0426
15	669.0937	4.606	Myricetin 3,7-diglucuronide	C_27_H_26_O_20_	670.1008
16	299.0404	4.944	Mumefural	C_12_H_12_O_9_	300.0477
17	313.0562	5.082	Salicyl acyl glucuronide	C_13_H_14_O_9_	314.0636
18	469.0044	5.157	Sanguisorbic acid dilactone	C_21_H_10_O_13_	470.0117
19	181.0144	5.345	2-Hydroxyisophthalic acid	C_8_H_6_O_5_	182.0217
20	313.0562	5.621	Salicyl phenolic glucuronide	C_13_H_14_O_9_	314.0634
21	285.0612	5.846	Uralenneoside	C_12_H_14_O_8_	286.0684
22	359.0979	6.523	6′-Methoxypolygoacetophenoside	C_15_H_2_0O_10_	360.1052
23	541.0258	6.999	Punicacortein D	C_48_H_28_O_30_	1084.0660
24	1083.0581	7.024	Punicalagin	C_48_H_28_O_30_	1084.0653
25	347.0770	7.162	Alpha-(1,2-dihydroxyethyl)-1,2,3,4-tetrahydro-7-hydroxy-9-methoxy-3,4-dioxocyclopenta[c] [1]benzopyran-6-acetaldehyde	C_17_H_16_O_8_	348.0842
26	483.0776	7.324	1,2′-di-O-galloylhamamelofuranose	C_20_H_2_0O_14_	484.0848
27	403.1240	7.625	Oleoside 11-methyl ester	C_17_H_24_O_11_	404.1313
28	220.0616	7.751	Methyl dioxindole-3-acetate	C_11_H_11_NO_4_	221.0689
29	321.0253	7.951	Digallate	C_14_H_10_O_9_	322.0325
30	183.0297	8.126	Methyl 2,4,6-trihydroxybenzoate	C_8_H_8_O_5_	184.0370
31	467.1189	8.452	Leucodelphinidin 3-O-alpha-L-rhamnopyranoside	C_21_H_24_O_12_	468.1261
32	219.0293	8.552	Purpurogallin	C_11_H_8_O_5_	220.0367
33	635.0885	9.003	3-O-galloylhamamelitannin	C_27_H_24_O_18_	636.0958
34	295.0456	9.304	cis-Coutaric acid	C_13_H_12_O_8_	296.0528
35	651.0831	9.454	Amlaic acid	C_27_H_24_O_19_	652.0903
36	477.0671	9.479	Quercetin 3′-O-glucuronide	C_21_H_18_O_13_	478.0743
37	633.0744	10.006	Pterocaryanin B	C_27_H_22_O_18_	634.0813
38	785.0837	10.970	Sanguiin H1	C_34_H_26_O_22_	786.0908
39	515.1912	11.133	Spicatin	C_27_H_32_O_10_	516.1983
40	371.0976	11.734	Dihydroferulic acid 4-O-glucuronide	C_16_H_20_O_10_	372.1049
41	249.0399	12.035	2-hydroxy-6-oxo-6-(2-hydroxyphenoxy)-hexa-2,4-dienoate	C_12_H_10_O_6_	250.0472
42	247.0247	12.235	7-deshydroxypyrogallin-4-carboxylic acid	C_12_H_8_O_6_	248.0320
43	447.0927	12.436	1,2,6,8-tetrahydroxy-3-methylanthraquinone 2-O-b-D-glucoside	C_21_H_20_O_11_	448.0999
44	239.0557	12.561	(1R,6R)-6-hydroxy-2-succinylcyclohexa-2,4-diene-1-carboxylate	C_11_H_12_O_6_	240.0629
45	600.9885	12.724	Diellagilactone	C_28_H_10_O_16_	601.9956
46	465.1027	13.238	(−)-epicatechin 7-O-glucuronide	C_21_H_22_O_12_	466.1100
47	197.0455	13.313	3,4-O-dimethylgallic acid	C_9_H_10_O_5_	198.0527
48	937.0934	13.338	Punicafolin	C_41_H_30_O_26_	938.1004
49	953.0890	13.664	Isoterchebin	C_41_H_30_O_27_	954.0963
50	787.0992	14.628	1,2′,3,5-tetra-O-galloylhamamelofuranose	C_34_H_28_O_22_	788.1064
51	609.1454	14.904	Luteolin 6-C-glucoside 8-C-arabinoside	C_27_H_30_O_16_	610.1527
52	431.0976	14.966	Isovitexin	C_21_H_20_O_10_	432.1048
53	300.9989	15.217	Ellagic acid	C_14_H_6_O_8_	302.0061
54	421.0772	15.831	Isomangiferin	C_19_H_18_O_11_	422.0844
55	491.0822	15.994	Isorhamnetin 4′-O-glucuronide	C_22_H_20_O_13_	492.0895
56	357.1183	16.469	Phlorisobutyrophenone 2-glucoside	C_16_H_22_O_9_	358.1256
57	955.1049	16.633	Chebulinic acid	C_41_H_32_O_27_	956.1120
58	355.1027	17.071	1-O-2′-hydroxy-4′-methoxycinnamoyl-b-D-glucose	C_16_H_20_O_9_	356.1100
59	261.0403	17.422	2-Acetyl-5,8-dihydroxy-3-methoxy-1,4-naphthoquinone	C_13_H_10_O_6_	262.0475
60	435.0930	17.497	Taxifolin 3-arabinoside	C_20_H_20_O_11_	436.1001
61	207.0660	18.725	Sinapyl aldehyde	C_11_H_12_O_4_	208.0732
62	259.0243	18.988	Urolithin D	C_13_H_8_O_6_	260.0316
63	243.0655	19.226	3-Desmethyl-5-deshydroxyscleroin	C_14_H_12_O_4_	244.0727
64	331.0817	19.552	2′,3,5-trihydroxy-5′,7-dimethoxyflavanone	C_17_H_16_O_7_	332.0889
65	461.0723	20.529	3-Methylellagic acid 8-rhamnoside	C_21_H_18_O_12_	462.0796
66	287.0557	21.180	3′,4′,5,7-tetrahydroxyisoflavanone	C_15_H_12_O_6_	288.0630
67	217.0503	21.305	Piperic acid	C_12_H_10_O_4_	218.0576
68	355.0450	22.182	Grevilline C	C_18_H_12_O_8_	356.0525
69	573.0875	23.886	Mangiferin 6′-gallate	C_26_H_22_O_15_	574.0947
70	303.0508	23.987	Pratenol B	C_15_H_12_O_7_	304.0580
71	461.1086	25.941	Rhamnetin 3-rhamnoside	C_22_H_22_O_11_	462.1159
72	285.0403	27.494	Luteolin	C_15_H_10_O_6_	286.0476
73	147.0449	27.720	Trans-cinnamic acid	C_9_H_8_O_2_	148.0522
74	301.0348	27.795	Hieracin	C_15_H_10_O_7_	302.0421
75	567.1134	28.834	Chrysophanol 8-(6-galloylglucoside)	C_28_H_24_O_13_	568.1206
76	329.0300	28.948	2,8-di-O-methylellagic acid	C_16_H_10_O_8_	330.0373
77	695.4003	31.203	Glucosyl passiflorate	C_37_H_60_O_12_	696.4074
78	613.1187	31.278	6-cinnamoyl-1,2-digalloylglucose	C_29_H_26_O_15_	614.1259
79	329.2329	33.307	9S,10S,11R-trihydroxy-12Z-octadecenoic acid	C_18_H_34_O_5_	330.2402
80	287.2223	35.186	9,10-dihydroxy-hexadecanoic acid	C_16_H_32_O_4_	288.2295
81	503.3372	35.262	(3 beta, 19 alpha)-3,19,23,24-tetrahydroxy-12-oleanen-28-oic acid	C_30_H_48_O_6_	504.3444
82	273.0402	35.449	1,3,6-trihydroxy-5-methoxyxanthone	C_14_H_10_O_6_	274.0474
83	343.0456	36.565	Aflatoxin GM1	C_17_H_12_O_8_	344.0529

**Table 2:** Tentative identification of the chemical constituents from JPT by LC-QTOF-MS analysis.

No.	M/Z	RT (min)	Compounds	Formula	Molecular weight (g/mol)	
1	333.0589	1.719	2-(beta-D-glucosyl)-sn-glycerol 3-phosphate	C_9_H_19_O_11_P	334.0663	
2	191.0560	1.794	Quinic acid	C_7_H_12_O_6_	192.0633	
3	181.0721	1.819	D-sorbitol	C_6_H_14_O_6_	182.0793	
4	209.0304	1.932	Galactaric acid	C_6_H_10_O_8_	210.0377	
5	191.0204	1.970	Glucaric acid lactone	C_6_H_8_O_7_	192.0277	
6	361.0413	2.045	2-O-galloylgalactaric acid	C_13_H_14_O_12_	362.0486	
7	355.0311	2.809	(+)-chebulic acid	C_14_H_12_O_11_	356.0383	
8	169.0146	3.410	Gallic acid	C_7_H_6_O_5_	170.0218	
9	243.0510	3.548	1-O-galloylglycerol	C_10_H_12_O_7_	244.0582	
10	311.0409	3.699	cis-caffeoyl tartaric acid	C_13_H_12_O_9_	312.0484	
11	343.0306	3.786	5-O-galloyl-1,4-galactarolactone	C_13_H_12_O_11_	344.0379	
12	325.0566	3.899	Fertaric acid	C_14_H_14_O_9_	326.0639	
13	651.1197	3.949	Chrysoeriol 4′,7-diglucuronide	C_28_H_28_O_18_	652.1268	
14	191.0349	4.300	5,7-dihydroxy-4-methylcoumarin	C_10_H_8_O_4_	192.0422	
15	235.0246	4.325	2-hydroxy-3-carboxybenzalpyruvate	C_11_H_8_O_6_	236.0320	
16	933.0620	4.350	2-O-galloylpunicalin	C_41_H_26_O_26_	934.0690	
17	265.0354	4.488	2-O-p-coumaroyltartronic acid	C_12_H_10_O_7_	266.0428	
18	669.0933	4.676	Myricetin 3,7-diglucuronide	C_27_H_26_O_20_	670.1007	
19	299.0408	4.826	Mumefural	C_12_H_12_O_9_	300.0481	
20	469.0045	5.202	Sanguisorbic acid dilactone	C_21_H_10_O_13_	470.0117	
21	181.0142	5.352	2-Hydroxyisophthalic acid	C_8_H_6_O_5_	182.0215	
22	483.0783	5.603	1,2′-di-O-galloylhamamelofuranose	C_20_H_2_0O_14_	484.0854	
23	313.0561	5.628	Salicyl phenolic glucuronide	C_13_H_14_O_9_	314.0634	
24	234.0407	5.753	3,4-dihydro-7-methoxy-2-methylene-3-oxo-2H-1,4-benzoxazine-5-carboxylic acid	C_11_H_9_NO_5_	235.0481	
25	285.0611	5.853	Uralenneoside	C_12_H_14_O_8_	286.0683	
26	403.1238	6.191	Oleoside 11-methyl ester	C_17_H_24_O_11_	404.1310	
27	359.0984	6.505	6′-methoxypolygoacetophenoside	C_15_H_20_O_10_	360.1056	
28	1083.0578	7.031	Punicalagin	C_48_H_28_O_30_	1084.065	
29	541.0258	7.081	Punicacortein D	C_48_H_28_O_30_	1084.066	
30	347.0770	7.231	Alpha-(1,2-dihydroxyethyl)-1,2,3,4-tetrahydro-7-hydroxy-9-methoxy-3,4-dioxocyclopenta[c] [1]benzopyran-6-acetaldehyde	C_17_H_16_O_8_	348.0842	
31	220.0614	7.795	Methyl dioxindole-3-acetate	C_11_H_11_NO_4_	221.0686	
32	321.0251	7.945	Digallate	C_14_H_10_O_9_	322.0323	
33	1083.1151	8.159	Putranjivain A	C_46_H_36_O_31_	1084.122	
34	467.1191	8.409	Leucodelphinidin 3-O-alpha-L-rhamnopyranoside	C_21_H_24_O_12_	468.1263	
35	219.0295	8.559	Purpurogallin	C_11_H_8_O_5_	220.0368	
36	635.0882	9.023	3-O-galloylhamamelitannin	C_27_H_24_O_18_	636.0955	
37	295.0453	9.336	cis-Coutaric acid	C_13_H_12_O_8_	296.0525	
38	651.0832	9.462	Amlaic acid	C_27_H_24_O_19_	652.0905	
39	477.0671	9.486	Quercetin 3′-O-glucuronide	C_21_H_18_O_13_	478.0742	
40	633.0744	10.013	Pterocaryanin B	C_*2*7_H_22_O_18_	634.0812	
41	785.0834	10.990	Sanguiin H1	C_34_H_26_O_22_	786.0904	
42	515.1912	11.165	Spicatin	C_27_H_32_O_10_	516.1984	
43	371.0975	11.742	Dihydroferulic acid 4-O-glucuronide	C_16_H_20_O_10_	372.1047	
44	935.0773	11.766	1-O-galloylpedunculagin	C_41_H_28_O_26_	936.0843	
45	249.0399	12.067	2-hydroxy-6-oxo-6-(2-hydroxyphenoxy)-hexa-2,4-dienoate	C_12_H_10_O_6_	250.0472	
46	247.0246	12.243	7-deshydroxypyrogallin-4-carboxylic acid	C_12_H_8_O_6_	248.0320	
47	447.0926	12.443	1,2,6,8-tetrahydroxy-3-methylanthraquinone 2-O-b-D-glucoside	C_21_H_20_O_11_	448.0998	
48	239.0556	12.568	(1R,6R)-6-hydroxy-2-succinylcyclohexa-2,4-diene-1-carboxylate	C_11_H_12_O_6_	240.0629	
49	600.9885	12.731	Diellagilactone	C_28_H_10_O_16_	601.9956	
50	197.0456	13.320	3,4-O-dimethylgallic acid	C_9_H_10_O_5_	198.0528	
51	937.0937	13.345	Punicafolin	C_41_H_30_O_26_	938.1007	
52	953.0890	13.734	Isoterchebin	C_41_H_30_O_27_	954.0963	
53	787.0990	14.723	1,2′,3,5-tetra-O-galloylhamamelofuranose	C_34_H_28_O_22_	788.1064	
54	431.0975	14.899	Isovitexin	C_21_H_2_0O_10_	432.1047	
55	300.9991	15.124	Ellagic acid	C_14_H_6_O_8_	302.0064	
56	421.0773	15.851	Isomangiferin	C_19_H_18_O_11_	422.0844	
57	491.0824	15.926	Isorhamnetin 4′-O-glucuronide	C_22_H_20_O_13_	492.0897	
58	463.0874	15.976	Quercetin 3-galactoside	C_21_H_20_O_12_	464.0947	
59	303.0503	16.577	(±)-taxifolin	C_15_H_12_O_7_	304.0576	
60	955.1041	16.628	Chebulinic acid	C_41_H_32_O_27_	956.1113	
61	355.1027	17.078	1-O-2′-hydroxy-4′-methoxycinnamoyl-b-D-glucose	C_16_H_20_O_9_	356.1100	
62	939.1095	17.204	1,2,3,4,6-pentakis-O-galloyl-beta-D-glucose	C_41_H_32_O_26_	940.1166	
63	435.0928	17.417	Taxifolin 3-arabinoside	C_20_H_20_O_11_	436.0999	
64	261.0402	17.429	2-Acetyl-5,8-dihydroxy-3-methoxy-1,4-naphthoquinone	C_13_H_10_O_6_	262.0474	
65	276.0507	17.579	2-phthalimidoglutaric acid	C_13_H_11_NO_6_	277.0579	
66	207.0659	18.720	Sinapyl aldehyde	C_11_H_12_O_4_	208.0731	
67	259.0242	19.083	Urolithin D	C_13_H_8_O_6_	260.0316	
68	461.0721	19.634	3-Methylellagic acid 8-rhamnoside	C_21_H_18_O_12_	462.0793	
69	287.0556	21.163	3′,4′,5,7-tetrahydroxyisoflavanone	C_15_H_12_O_6_	288.0629	
70	217.0502	21.313	Piperic acid	C_12_H_10_O_4_	218.0575	
71	303.0508	24.019	Pratenol B	C_15_H_12_O_7_	304.0580	
72	673.2124	24.370	Premithramycin A2′	C_33_H_38_O_15_	674.2196	
73	461.1084	25.961	Rhamnetin 3-rhamnoside	C_22_H_22_O_11_	462.1157	
74	285.0401	27.539	Luteolin	C_15_H_10_O_6_	286.0474	
75	147.0449	27.740	trans-Cinnamic acid	C_9_H_8_O_2_	148.0522	
76	301.0352	27.890	Hieracin	C_15_H_10_O_7_	302.0424	
77	295.0970	28.654	1-(4-hydroxy-3-methoxyphenyl)-5-(4-hydroxyphenyl)-1,4-pentadien-3-one	C_18_H_16_O_4_	296.1043	
78	329.0301	28.967	2,8-di-O-methylellagic acid	C_16_H_10_O_8_	330.0373	
79	695.4004	31.235	Glucosyl passiflorate	C_37_H_60_O_12_	696.4074	
80	613.1187	31.310	6-cinnamoyl-1,2-digalloylglucose	C_29_H_26_O_15_	614.1260	
81	287.2224	35.156	9,10-dihydroxy-hexadecanoic acid	C_16_H_32_O_4_	288.2296	
82	503.3374	35.294	(3 beta, 19 alpha)-3,19,23,24-tetrahydroxy-12-oleanen-28-oic acid	C_30_H_48_O_6_	504.3445	
83	273.0399	35.519	1,3,6-trihydroxy-5-methoxyxanthone	C_14_H_10_O_6_	274.0472	
84	343.0459	36.572	Aflatoxin GM1	C_17_H_12_O_8_	344.0531	

**Table 3:** Percentage parasitemia and suppression of TSM and JPT recipes.

Group	Dose (mg/kg)	% parasitemia	% suppression
Not infected + PBS	—	—	—

Infected + PBS	—	18.93 ± 0.81	—

Infected + CQ	25	0^a^	100^a^

Infected + TSM	200	9.02 ± 0.72^a,b^	52.35 ± 3.82^a,b^
	400	8.02 ± 0.77^a,b^	57.63 ± 4.09^a,b^
	600	6.24 ± 0.57^a,b^	67.02 ± 2.99^a,b^

Infected + JPT	200	8.62 ± 1.70^a,b^	54.46 ± 8.97^a,b^
	400	6.67 ± 0.72^a,b^	64.79 ± 3.83^a,b^
	600	3.91 ± 1.11^a,c,h^	79.34 ± 5.86^a,c,h^

Data are presented as mean ± SEM (*n* = 5 per group). Differences were considered statistically significant at *p* < 0.05. ^a^compared with the negative control group receiving PBS, ^b^compared with the positive control group receiving CQ, ^c^compared with TSM 200 mg/kg, ^d^compared with TSM 400 mg/kg, ^e^compared with TSM 600 mg/kg, ^f^compared with JPT 200 mg/kg, ^g^compared with JPT 400 mg/kg, and ^h^compared with JPT 600 mg/kg.

**Table 4:** Body weight and organ weight in acute toxicity test.

Group	Body weight (g)		% increase of body weight	Relative weight (g)	
	Day 0	Day 14		Liver	Kidney
PBS	37.71 ± 0.56	41.68 ± 0.87	10.54 ± 1.51	6.96 ± 0.73	1.89 ± 0.12
TSM	35.35 ± 1.06	38.41 ± 1.31	8.68 ± 1.02	5.74 ± 0.32	1.73 ± 0.80
JPT	35.62 ± 0.74	38.78 ± 0.90	8.88 ± 1.37	5.82 ± 0.47	1.89 ± 0.92

All values are expressed as mean ± SEM. There were no statistically significant differences at *p* < 0.05.

**Table 5:** Biochemical profiles of the liver and kidney function in acute toxicity test.

Group	BUN (mg/dL)	CREA (mg/dL)	AST (U/L)	ALT (U/L)	ALP (U/L)
PBS	24.60 ± 0.40	0.16 ± 0.00	113.60 ± 13.72	32.00 ± 1.97	119.60 ± 18.20
TSM	24.20 ± 1.43	0.16 ± 0.01	116.40 ± 16.46	36.40 ± 6.02	117.40 ± 15.78
JPT	21.60 ± 0.81	0.18 ± 0.01	92.80 ± 8.52	29.80 ± 2.35	104.80 ± 7.34

All values are expressed as mean ± SEM. There were no statistically significant differences at *p* < 0.05.

## Data Availability

The data associated with this study are included within the published article. Additional files are available from corresponding authors upon request.
